# Data on interleukin (IL)-2- and IL-15-dependent changes in IL-2R*β* and IL-2Rγ complexes

**DOI:** 10.1016/j.dib.2017.02.030

**Published:** 2017-03-02

**Authors:** Nerea Osinalde, Virginia Sánchez-Quiles, Blagoy Blagoev, Irina Kratchmarova

**Affiliations:** a Department of Biochemistry and Molecular Biology, University of the Basque Country UPV/EHU, 01006 Vitoria-Gasteiz, Spain; b Molecular Oncology Group, UMR 144 CNRS, Curie Institute, 26, rue d’Ulm, 75248 Paris, France; c Department of Biochemistry and Molecular Biology, University of Southern Denmark, Odense M, Denmark

**Keywords:** SILAC, Interleukin, Cell signaling, Phosphotyrosine, T-lymphocytes, Interactome

## Abstract

We provide detailed datasets from our analysis of the proteins that associate with IL-2Rβ and IL-2Rγ in T-cells stimulated with IL-2 or IL-15 compared with resting T-cells, as identified by SILAC-based quantitative proteomics. We also include quantitative data regarding site-specific phosphorylation events observed both in IL-2Rβ and IL-2Rγ. Moreover, we provide results demonstrating the specific protein recruitment capacity of four of those site-specific phosphorylations. The proteomics and phosphoproteomics data described in this article is associated with a research article entitled “Characterization of receptor-associated protein complex assembly in Interleukin (IL)-2- and IL-15-activated T-lymphocytes” (Osinalde et al., 2016 [1]). The mass spectrometry data have been deposited to the ProteomeEXchange Constorium with the identifier PXD002386.

**Specifications Table**

**Table:** 

Subject area	*Immunology*
More specific subject area	*Protein-protein interaction, site-specific phosphorylation, phosphosite-dependent interaction*
Type of data	*Mass Spectrometry (MS) data*
How data was acquired	*MS data was acquired in a Q-Exactive (Thermo) mass spectrometer and Velos Orbitrap MS system (Thermo).*
Data format	Raw (*raw), excel files (.xlsx), figures
Experimental factors	***For IL-2R interactome analyses:** Kit225 T-cells were grown in light (Arg0/Lys0), medium (Arg6/Lys4) and heavy (Arg10/Lys8) media. Differentially SILAC-labeled T-cells were kept unstimulated, treated with IL-2 or stimulated with IL-15, respectively prior to cell lysis.*
	***For peptide pull-down analyses:** Kit225 T-cells were grown in light (Arg0/Lys0) and heavy (Arg10/Lys8) media.*
Experimental features	***For IL-2R interactome and phosphorylation analyses**, after stimulation, cells were lysed and protein extracts derived from the three different experimental conditions were combined and affinity-purified using specific antibodies against IL-2R beta or gamma subunits. Immune complexes were fractionated on a SDS-PAGE and in-gel digested using trypsin. Resulting peptides were either directly analyzed by LC-MS/MS or enriched in phosphorylated peptides using TiO*_*2*_*beads prior to MS analysis using a QE-Exactive MS instrument.*
	***For peptide pull-down analyses,** differentially labeled cells were lysed and separately incubated with the unmodified and phosphorylated version of the peptide used as bait. Then precipitated proteins were on-bead digested with LysC and subsequently with trypsin and resulting peptides were analyzed by LC-MS/MS in a Velos Orbitrap MS system.*
Data source location	*Odense, Denmark*
Data accessibility	*Data are available in this article and deposited at ProteomeEXchange Constorium,*http://www.proteomexchange.org/.

**Value of the data**

• The study uncovers new IL-2- and IL-15-dependent interacting partners of IL-2Rβ and IL-2Rγ.
• This investigation provides unprecedented data regarding cytokine-dependent and –independent phosphorylation events occurring in IL-2R β and γ subunits.
• A large number of phosphosites corresponding to a wide range of proteins are reported.
• The data presented here underscores the capacity of certain cytokine-dependent phosphorylation sites localized on IL-2Rβ and IL-2Rγ to recruit downstream signaling molecules.
• Overall, the study provides novel insights into the early activation events following interleukin/receptor engagement in CD4^+^ T-lymphocytes.

## 1 Data

The data in this article show the effect of IL-2 and IL-15 stimulation on the phosphorylation state and interacting partners of IL-2Rβ and IL-2Rγ. Data on the capacity of selected phosphorylations to serve as anchoring sites and recruit proteins are also presented. In all the experiments a SILAC-based approach was followed in combination with LC-MS/MS and subsequent bioinformatic analyses.

## 2 Experimental design, materials and methods

### 2.1 Cell culture

For mass spectrometry (MS)-based analysis, human leukemic Kit225 T-cells, which depend on IL-2 [2], were grown in RPMI deficient in arginine and lysine supplemented with 10% dyalized serum (Gibco-Invitrogen), 1% Glutamax, 1% penicillin/streptomycin (p/s), 1% sodium pyruvate and 16 U/ml of recombinant human IL-2 (kindly provided by AIDS Research and Reference Reagent Program, Division of AIDS, NIAD, NIH, USA) at a density of 1×10^6^ cells/ml at 37°C and 5% CO_2_. Cells were SILAC labelled [3] by adding externally distinct isotopes of L-Arginine (18 µg/ml) and L-Lysine (730 µg/ml. *Light* cells were grown in the presence of L-Arginine (Arg0)/L-Lysine (Lys0) whereas *medium* and *heavy* cells were maintained in the presence of L-Arginine-^13^C_6_ (Arg6)/L-Lysine-^2^H_4_ (Lys4) and L-Arginine-^13^C_6_^15^N_4_ (Arg10)/L-Lysine-^13^C_6_^15^N_2_ (Lys8), respectively (Cambridge isotope Laboratories). In order to achieve complete labeling of the proteome, Kit225 T-cells were maintained in SILAC media for at least five cells divisions.

### 2.2 Cytokine stimulation and protein extraction

**Fig. 1:**
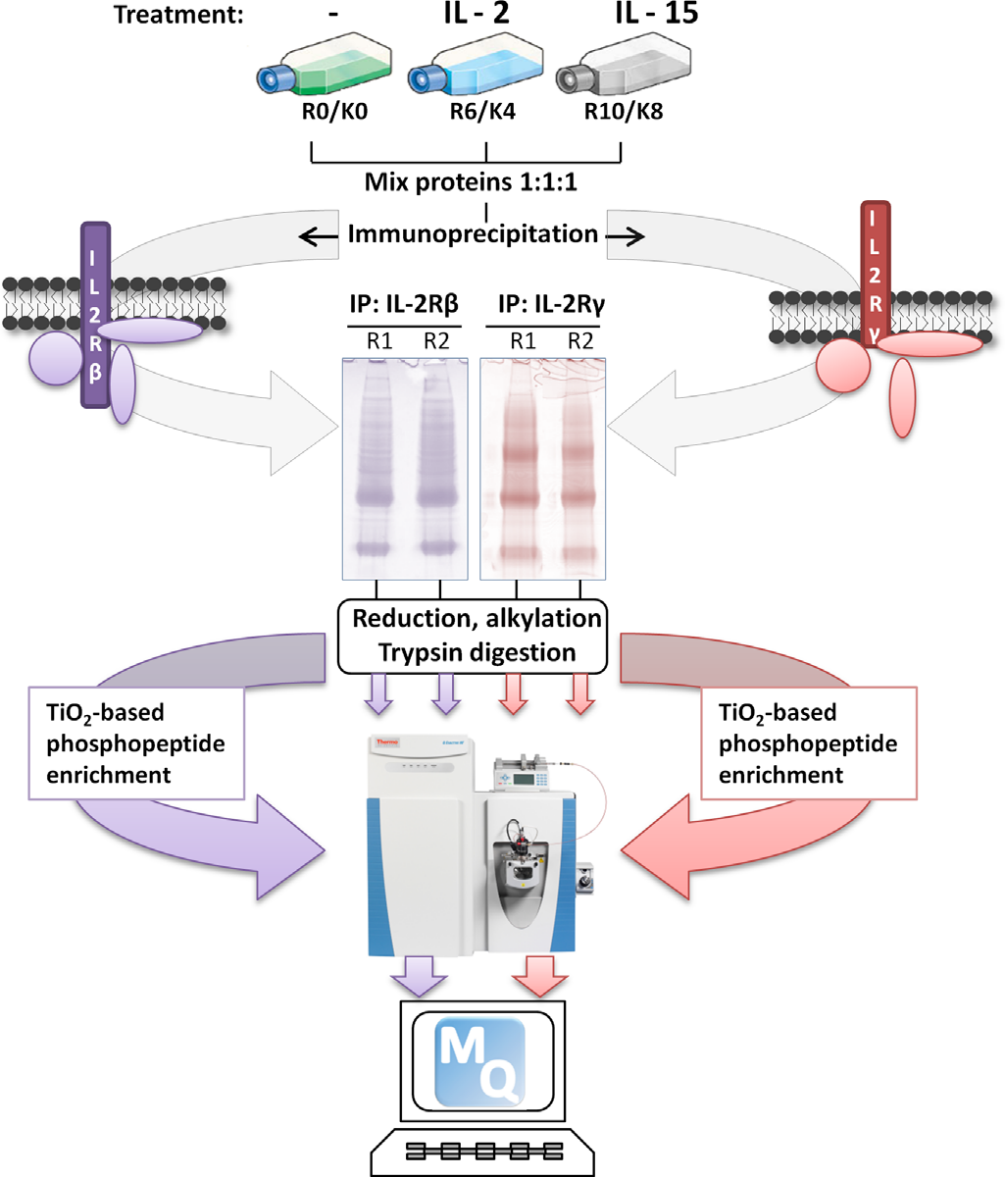
Experimental workflow for detection of cytokine-dependent and -independent interactors and phosphorylation events on IL–2R beta and gamma subunits.

To study the interactome of IL-2Rβ and IL-2Rγ in resting and interleukin-treated T-cells a triple SILAC-based MS strategy was carried out. Prior to the treatment, Kit225 T-cells were deprived with the cytokine for 48 h to synchronize them at G_1_ phase of the cell cycle and mimic resting T-lymphocytes. Subsequently, interleukin receptor complex assembly was induced by incubating medium- and heavy-labeled Kit225 T-cells (50×10^6^ cells/ml) with 200 U/ml of human recombinant IL-2 or IL-15 (Peprotech) respectively, for 5 min at 37 °C in a water bath. T-cells grown in light media were kept untreated and served as control (Fig. 1).

Four different peptide/phosphopeptide pull down assays were carried out. For each of them, a double SILAC experiment was perform in duplicate where Kit225 T-cells were grown either in light or heavy medium. Cells were starved for 2 days and then, the same procedure was applied to both cellular conditions: half of the cell population was kept unstimulated whereas the other half was treated with 200 U/ml IL-2 as mentioned above.

Interleukin stimulation was quenched by rapidly diluting cells on chilled PBS and washing them twice with ice-cold PBS. Differentially treated cells were lysed separately by incubating with rotation at 4 °C with co-immunoprecipitation buffer (25 mM TrisHCl pH 7.5, 100 mM NaCl, 1% NP-40, 1 mM sodium pervanadate, 5 mM beta-glycerophosphate, 5 mM NaF, complete protease inhibitor cocktail (complete tablets, Roche)) and lysates were then cleared by centrifugation (15,000*g*, 15 min at 4 °C). Protein concentrations of the soluble fractions were estimated using BCA protein assay kit (Pierce) following manufacturer׳s instructions.

### 2.3 Immunoprecipitation of IL-2R beta and gamma subunits

Light, medium and heavy labeled protein lysates derived from untreated, IL-2-stimulated and IL-15-stimulated T-cells respectively, were equally combined (in 1:1:1 ratio) according to their protein concentration and incubated with 50% slurry protein A-sepharose (GE Healthcare) for 1 h at 4 °C to eliminate proteins that bind non-specifically to the beads. Pre-cleared protein lysates were then incubated with antibodies against IL-2Rβ (sc-671) or IL-2Rγ (sc-667) at 4 °C for 4 h with rotation (Fig. 1). Then, immunoprecipitates were washed five times with lysis buffer and eluted by resuspending in Laemmli buffer and boiling at 95 °C for 5 min.

### 2.4 Fractionation and in-gel digestion of immunoprecipitated proteins

Proteins that were immunoprecipitated and hence, eluted were fractionated in two parallel lanes by one dimensional gel electrophoresis using a precast gradient NuPAGE 4–12% Bis-Tris Gel (Invitrogen) and stained with Colloidal Blue (Invitrogen). According to the bands visualized, the gel was cut into different size slices that were subsequently treated, as previously described in detail (our data in brief), with reducing and alkylating agents followed by trypsin so that the proteins undergo digestion. Resulting peptides were extracted from the gel by serial incubations with 100% acetonitrile (ACN) and 30% ACN/ 3% TFA at room temperature. Finally, recovered peptides were concentrated using a vacuum centrifuge before MS analysis (Fig. 1).

### 2.5 Enrichment of phosphorylated peptides using TiO_2_ beads

Enrichment of phosphopeptides was performed using titansphere beads [4] and following the protocol previously described in detail [4].

### 2.6 Peptide pull-down analysis

**Fig. 2:**
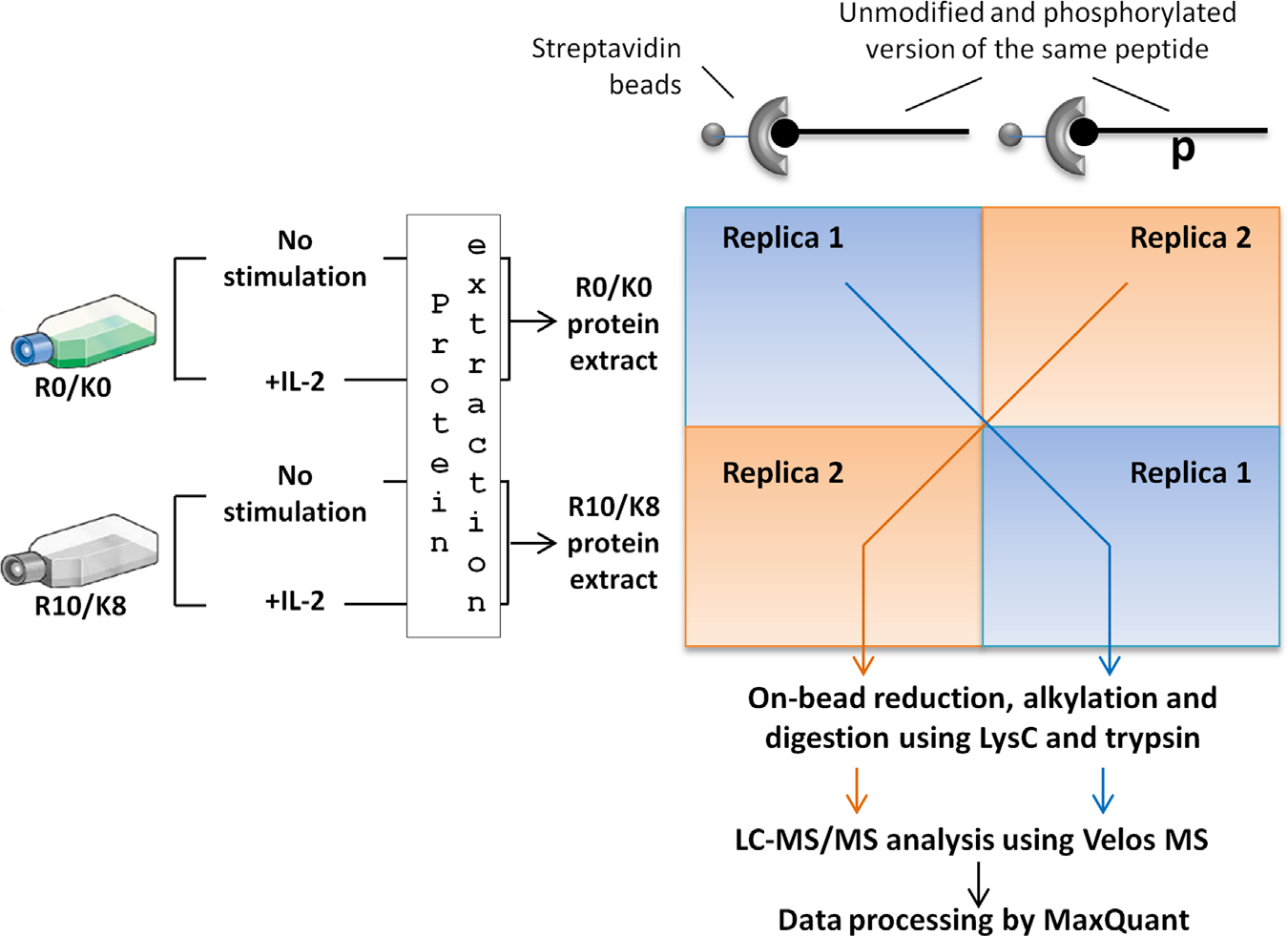
Schematic representation of the approach for characterization of recruitment capacity of cytokine-dependent phosphorylation sites detected in the activated IL-2R complex.

A series of N-terminally biotinylated phosphopeptide/peptide pairs were purchased from Peptide&Elephant: IL-2Rβ pS431/S431, SGDDLLLF**S**PSLLGG; IL-2Rβ pT476/T476, SGQPLGPP**T**PGVPDL; IL-2Rγ pY325/Y325 SGLAESLQPD**Y**SERLCL and IL-2Rγ pY357/Y357 SGPCNQHSP**Y**WAPPCY. The phosphorylated residue is highlighted in bold and underlined. Two pull down assays were carried out using each pair of peptides. In one of the replicas, protein extracts derived from T cells grown in light media were incubated with the unmodified peptide whereas proteins extracted from T cells grown in heavy media were incubated with the corresponding phosphorylated version of the same peptide. By contrast, in the second replica the labels were swapped so that heavy labeled proteins were incubated with the unmodified peptide and light proteins with the phosphopeptide (Fig. 2). It should be mentioned that in all the experiments, half of the T-cell population grown either in light or heavy media was kept unstimulated whereas the remaining half was treated with IL-2 for 5 min. Proteins were extracted by incubation in the co-immunoprecipitation buffer as described before (Section 2.2) and same amounts of differentially treated light and heavy protein extracts were combined. Subsequently, to remove sticky proteins from the samples light and heavy proteins were separately incubated with Dynabeads MyOne Streptavidine C1 (Thermo) for 2 h at 4 °C with rotation.

Meanwhile biotinylated peptides were coupled to Dynabeads MyOne Streptavidine C1 (Thermo) by incubating for at least 2 h at 4 °C in rotation. Unbound peptides were removed and pre-cleared protein lysates were added to the corresponding peptide or phosphopeptide and incubated for 2.5 h with rotation at 4 °C. Then, beads were subjected to 3 washes with complete lysis buffer and additional 3 washes with lysis buffer without detergent. Finally, beads loaded with the distinct version of the same peptide were combined.

### 2.7 On-bead digestion

Proteins precipitated with the phosphorylated or corresponding unmodified peptide were simultaneously subjected to on-bead digestion. For that purpose streptavidin beads loaded with the peptides and attached proteins were dissolved in urea buffer (8 M urea, 10 mM TrisHCl pH 7.5). Proteins were subjected to reduction and alkylation by 1 h incubation at room temperature using 1 mM DTT and 5.5 mM iodoacetamide, respectively. Then, Lys-C endoproteinase was added and the lysates were digested for 4 h at 37 °C. The resulting peptide mixtures were diluted with 10 mM TrisHCl pH 7.5 to achieve a final urea concentration below 2M prior to addition of trypsin that was incubated with the lysates at 37 °C over night. Trypsin activity was quenched by acidification using trifluoroacetic acid (TFA) to a final concentration of 1%. Prior to MS analysis, peptides were desalted and fractionated with C_18_ stage-tips by eluting with increasing acetonitrile concentration.

### 2.8 Mass spectrometry analysis

All procedures are described in detail in the associated research article [1].

### 2.9 Data analysis

MaxQuant software version 1.3.0.5 [5] was used to process the.raw files generated applying the parameters described before [6]. For protein identification, a minimum of 2 peptides, including 1 unique was required. Cysteine carbamidomethylation was set as fixed modification whereas M oxidation, N-terminal acetylation, NQ deamidation and STY phosphorylation were set as variable modifications.

**Fig. 3:**
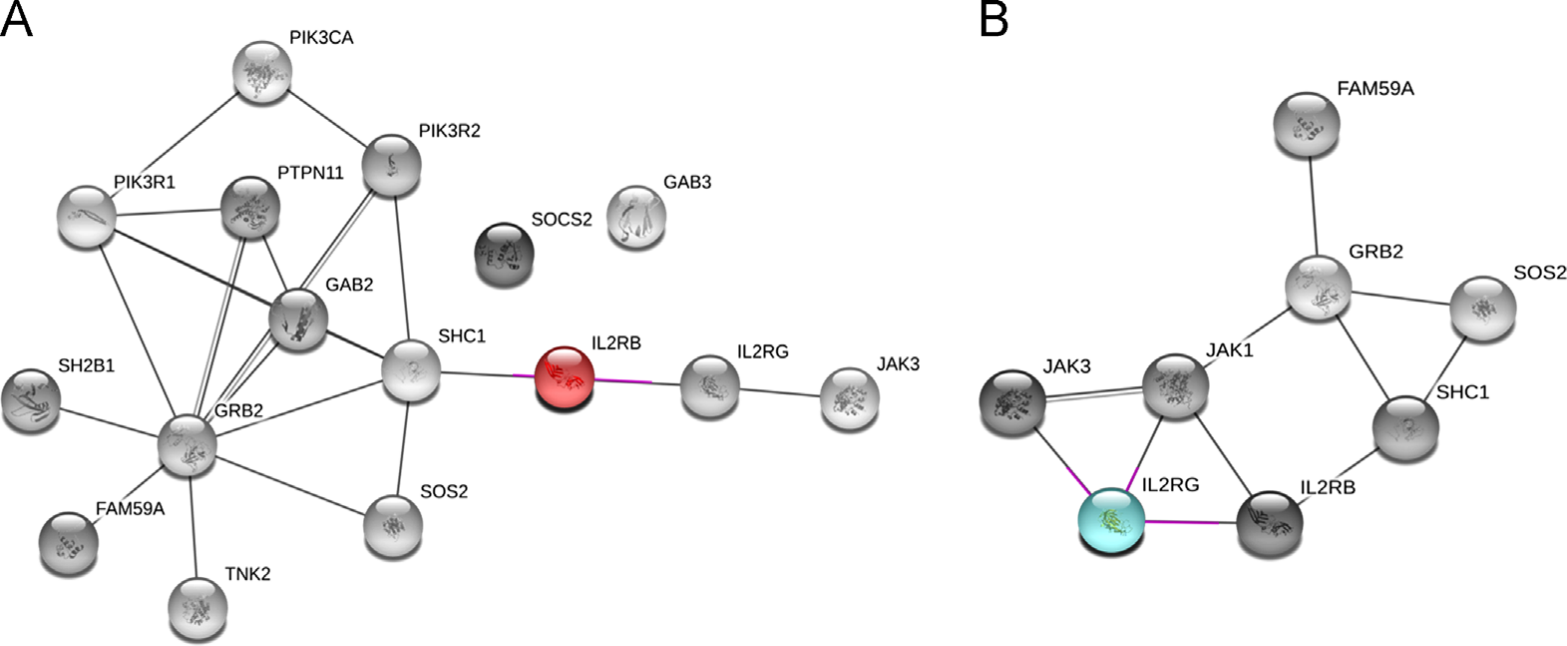
(A) IL-2Rβ- and (B) IL-2Rγ-centered protein-protein interaction network. The STRING search tool was used to generate a protein-protein interaction network using our dataset of cytokine-induced binding partners of the two IL2R signaling subunits. Only protein interactions that have an estimated probability of at least 0.7 based on experimental data are indicated.

**Fig. 4:**
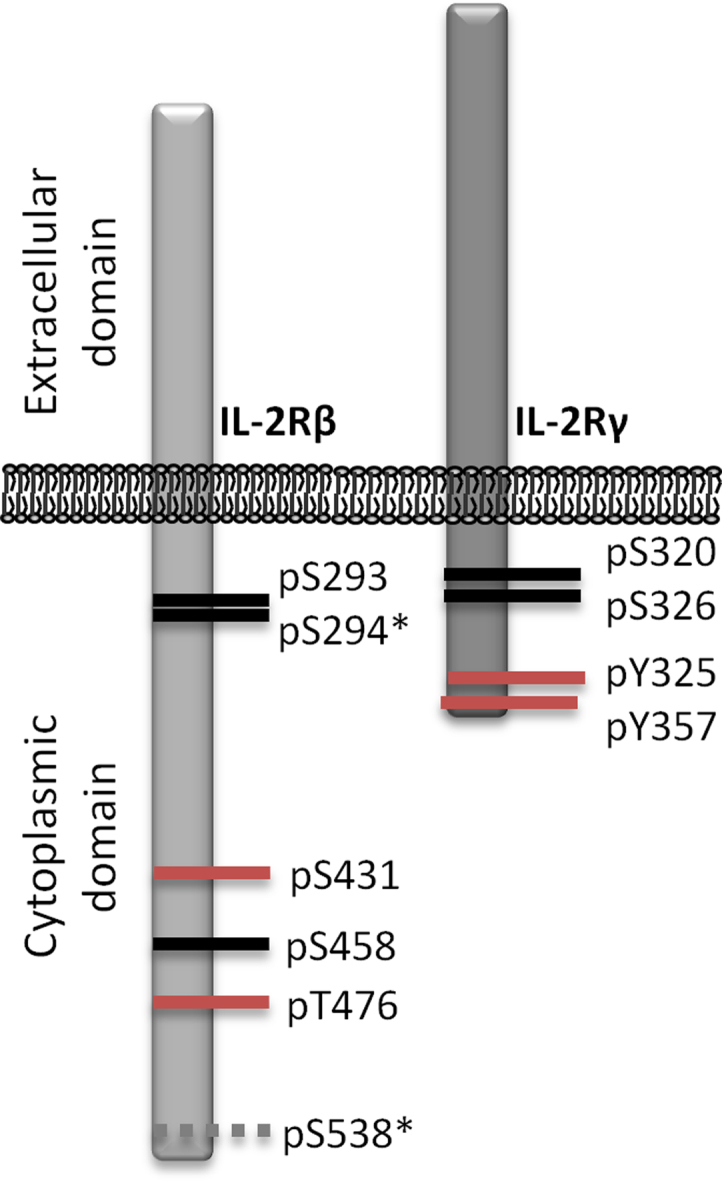
Site specific phosphorylations on IL-2R beta and gamma subunits. Phosphosites highlighted in red were found to be induced upon cytokine stimulation (cytokine/control ratio > 2) whereas the ones in black were not affected by the treatment. No SILAC ratio could be recorded for the pS538-containing peptide. *indicates that the phosphosite is not reported in the PhosphositePlus database.

Supplementary Tables 1 and 2 contain information about all identified and quantified proteins in the IL-2Rβ and IL-2Rγ immunoprecipitation experiments. Proteins displaying a cytokine (IL-2 or IL-15)/control > 2 and Significance B p value <0.001 were considered as cytokine-dependent binding partners of IL-2Rβ and/or IL-2Rγ. We used the STRING database [7] to evaluate the protein-protein interactions that were already reported within our list of cytokine-induced interactors of IL-2Rβ and IL-2Rγ (Fig. 3). As mentioned above part of the immune complexes containing IL-2Rβ and IL-2Rγ were subjected to trypsin digestion and TiO_2_-based enrichment of phosphopeptides prior to MS analysis. For the analysis of phosphopeptides a minimum localization probability of 0.75 was required. A total of 6 and 4 site specific phosphorylations corresponding to IL-2Rβ and IL-2Rγ respectively were detected (Fig. 4 and Supplementary Table 3). Moreover, Supplementary Table 4 contains information regarding additional phosphosites detected in this study.

**Fig. 5:**
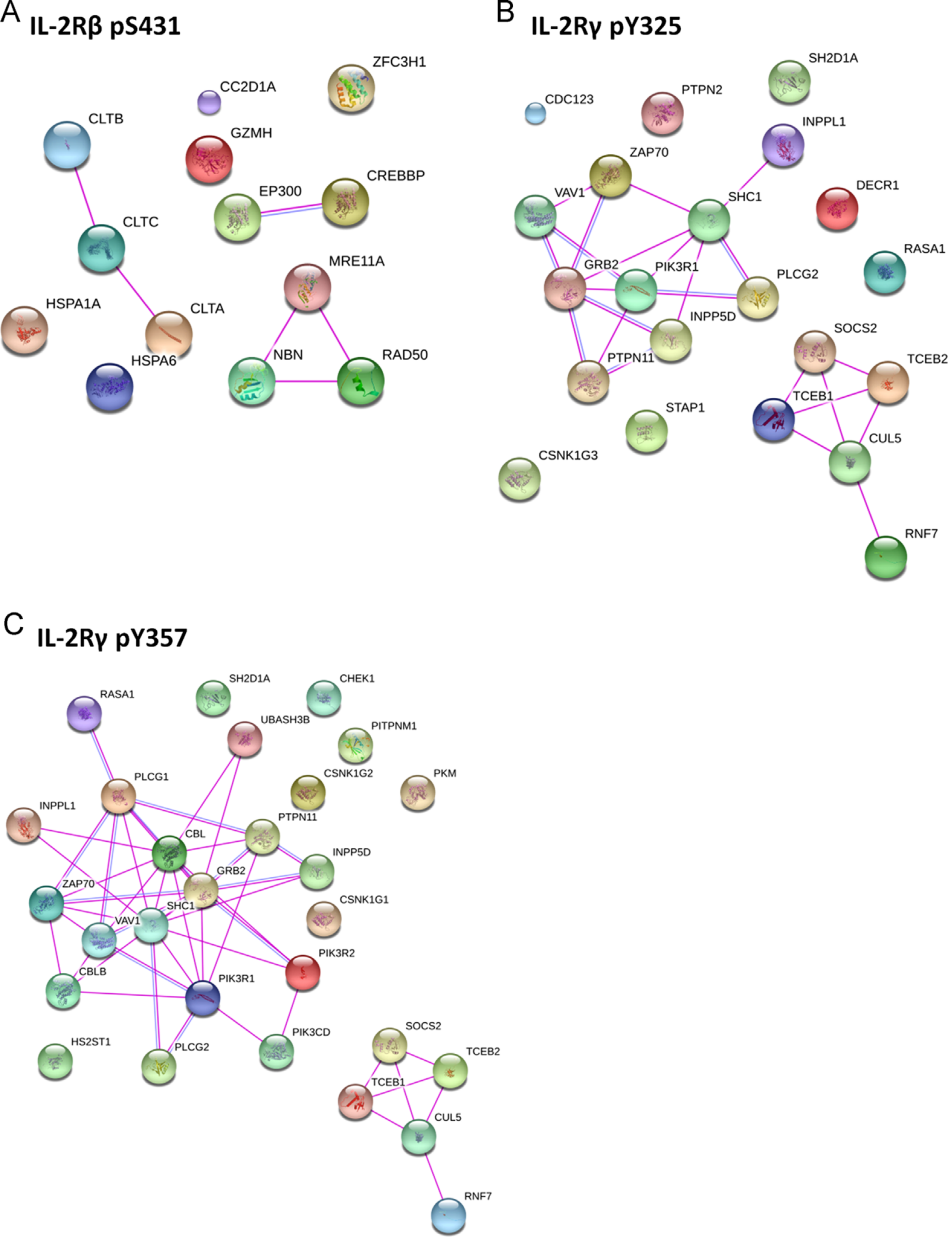
Protein interaction map of phosphosite-dependent proteins identified in the (A) IL-2Rβ pS431/S431, (B) IL-2Rγ pY325/Y325 and (C) IL-2Rγ pY357/Y357 pull down assays. The STRING search tool was used to generate the protein-protein interaction networks. Only protein interactions that have an estimated probability of at least 0.7 based on experimental data are indicated.

Supplementary Tables 5–8 contain information regarding all the proteins quantified in the two replicates of the 4 distinct peptide pull down assays performed. Proteins displaying a phosphopeptide/peptide ratio > 2 were considered as phosphosite-dependent binding partners of IL-2Rβ and/or IL-2Rγ. We used the STRING database [7] to evaluate the protein-protein interactions that were already reported within our list of phosphosite-dependent interactors of IL-2Rβ and IL-2Rγ (Fig. 5).
